# Modelling and Simulation of the Dynamics of the Antigen-Specific T Cell Response Using Variable Structure Control Theory

**DOI:** 10.1371/journal.pone.0166163

**Published:** 2016-11-18

**Authors:** Anet J. N. Anelone, Sarah K. Spurgeon

**Affiliations:** School of Engineering and Digital Arts, University of Kent, Canterbury, Kent, United Kingdom, CT2 7NT, United Kingdom; University of Glasgow, UNITED KINGDOM

## Abstract

Experimental and mathematical studies in immunology have revealed that the dynamics of the programmed T cell response to vigorous infection can be conveniently modelled using a sigmoidal or a discontinuous immune response function. This paper hypothesizes strong synergies between this existing work and the dynamical behaviour of engineering systems with a variable structure control (VSC) law. These findings motivate the interpretation of the immune system as a variable structure control system. It is shown that dynamical properties as well as conditions to analytically assess the transition from health to disease can be developed for the specific T cell response from the theory of variable structure control. In particular, it is shown that the robustness properties of the specific T cell response as observed in experiments can be explained analytically using a VSC perspective. Further, the predictive capacity of the VSC framework to determine the T cell help required to overcome chronic Lymphocytic Choriomeningitis Virus (LCMV) infection is demonstrated. The findings demonstrate that studying the immune system using variable structure control theory provides a new framework for evaluating immunological dynamics and experimental observations. A modelling and simulation tool results with predictive capacity to determine how to modify the immune response to achieve healthy outcomes which may have application in drug development and vaccine design.

## Introduction

This paper considers the extent to which variable structure control theory can be used to underpin the development of a modelling and simulation tool to analyse and tailor the dynamics of the specific immune response of T cells post infection. A Variable Structure Control System (VSCS) is a feedback system where the dynamic structure is changed to achieve performance requirements [1]. Switching between different dynamics is advantageous because the desirable properties of several subsystems can be combined so that the overall system possesses new and enhanced dynamical behaviour including properties that are not present in any of the individual subsystems alone. In particular, such VSCS are known to possess strong robustness properties in the presence of parameter uncertainty and disturbances [1, 2]. The theory of VSCS has been applied successfully to mechanical, electrical and chemical systems in the domain of engineering [1, 2]. Understanding of the quantitative and qualitative characteristics of the antigen-specific T cell response is important in immunology [3, 4]. An objective of this paper is to demonstrate the synergies between immunological dynamics and VSCS in order to deliver a new and constructive framework to assess the dynamics of health and disease.

The population of T lymphocytes consists of millions of clones characterized by their unique T cell receptor binding with antigen [5]. Each T cell clone is usually activated following the presentation of a specific antigen by Antigen Presenting Cells (APCs). The theory of the clonal expansion from [6] postulates that antigen-specific immune responses are produced by the proliferation of a small number of antigen-specific cells to a population sufficiently large to influence the progression of the specific pathogen. A number of practical studies have supported this postulate [3–5] because signals produced following interactions with self or foreign tissues induce variation in the behaviour and population dynamics of different immune cells and antibodies [4, 7, 8].

Experiments have demonstrated that after the recognition of bacteria, virus or infected cells, the antigen-specific response of T cells such as CD8+ T cells consists of three phases [7, 9, 10]. The activated T cell clones first exhibit expansion of their initial population so as to combat the pathogen. Next, the resultant large number of antigen-specific T cells undergoes contraction i.e cell death via apoptosis. Finally, the memory phase of the response consists of the differentiation of activated antigen-specific T cells into memory T cells [5]. Hence, the dynamics of the T cell response changes over a relatively short time (days) to induce variations in the population of the specific T cell clones so as to influence the performance of the immune system [4, 6, 9, 11].

Experimental data on the kinetics of the T cell response to different pathogens show two types of expansion dynamic [3, 9, 11, 12]. In some cases, the proliferation of activated T cells monitors and follows the concentration of pathogen [13, 14]. In other cases, an antigen independent expansion dynamic in which the proliferation of activated T cells continues after the infection is cleared is observed [7, 9, 15]. A linear, a Michaelis-Menten, a sigmoidal and a step-like immune response function have been constructed to model and investigate the antigen dependent expansion phase [16, 17]. The antigen independent expansion phase is often encompassed in a time based on/off immune response function leading to a piecewise linear system [7, 18, 19]. The effects of these candidate immune response functions have been reviewed from a system immunology perspective [3, 9, 12] and from a control engineering view-point in [20]. It has been deduced that the immune response function is a state feedback mechanism which influences the stability, the performance, the transient dynamics and the steady-state population of the immune system in both health and disease.

It will be seen that the different mathematical approaches proposed to model the dynamics of the antigen-specific response of T cells [3, 13, 21] can be unified into a single framework by adopting a variable structure control paradigm. A number of approaches have used saturation functions to prescribe the required changes in the T cell population [3, 13] and the work in [9] has referred to the immune response program as a switching process which governs the dynamic response of T cells post infection and have investigated the characteristics of different candidate immune response functions. From the viewpoint of variable structure control, the use of saturation functions to implement switched control algorithms is commonplace and the relationships between the two approaches are well understood.

Control theory has previously been used to investigate the dynamics of the immune response during health and infection where the resulting dynamics has been considered as a closed-loop system [20, 22, 23]. Contributions are of two types, where either the personal immune response function is considered as a control or the effect of drug treatment is formulated as an outer-loop control. For instance, in models of the dynamics of the Human Immunodeficiency Virus (HIV), Model Predictive Control (MPC) has been applied to propose an antiretroviral treatment regime to enhance the immune response [24, 25]. In [23] it has been demonstrated that the immune system can be regarded as a decentralised control system because the local interactions between individual immune cells in the presence of pathogen generate the population dynamics of the antigen-specific response of T cells. Moreover, this induced T cell population dynamics is found to mimic an on/off feedback control [23]. Preliminary work linking VSC theory and immunology has modelled the immune response of T cells to self antigen [20]. Control analysis has revealed that the performance of the immune response function exhibits robustness i.e insensitivity to variation in some metabolic rates and processes [16, 20, 26], under appropriate conditions.

As well as providing a modelling framework to represent the population dynamics of the specific T cell response, the predictive capacity of the variable structure control framework will also be demonstrated by considering experimental results on Lymphocytic Choriomeningitis Virus (LCMV) infection in mice. Experimental studies on LCMV infection in [4, 16, 27] have provided kinetic information on the variation over time of the number of CD8+ T cells in the spleen following acute and chronic infection. Following infection with a large viral dose, it has been seen that the immune response of memory CD8+ T cells is impaired due to high virus titers and antigen persistence [4]. Experimental studies have found that initiating a concurrent LCMV-specific CD4+ T cell response alongside the LCMV-specific CD8+ T cell response leads to a larger and sustained expansion of secondary effector CD8+ T cells. As discussed in [4], there is a need to better understand this phenomenon known as CD4+ T cell help because it has the potential to be an important element in vaccine design. To date, the molecular and cellular mechanism realising CD4+ T cell help are unclear and remain the subject of active research. Here analytical tools from VSC theory can be applied to deliver dynamical insights on the CD4+ T cell help mechanism.

## Materials and Methods

### System model

Consider the general model of the immune response as presented in [3] where the dynamical equations are given by

$$\begin{array}{ccc} \frac{dB}{dt} & = & rB-kBA \end{array}$$ (1)

$$\begin{array}{ccc} \frac{dN}{dt} & = & \sigma +r_{N}-a_{N}F\left(t\right)N-d_{N}N \end{array}$$ (2)

$$\begin{array}{ccc} \frac{dA}{dt} & = & F\left(t\right)\left(a_{N}N+a_{M}M+\rho A\right)-\left(1-F\left(t\right)\right)\left(mA\right)-d_{A}A \end{array}$$ (3)

$$\begin{array}{ccc} \frac{dM}{dt} & = & \left(1-F\left(t\right)\right)mA+r_{M}M-a_{M}F\left(t\right)M-d_{M}M \end{array}$$ (4)

The system in Eqs (1)–(4) describes the variation with time of the population of specific T cells responding to an exponentially growing pathogen. A description of the terms of the model Eqs (1)–(4) is provided in Tables 1 and 2. The likely range of the parameters given in Table 2 is obtained from the work in [3, 16, 18]. The function 0≤F(t)≤1 is an activation function prescribing the dynamical behaviour of the immune response in the presence of the pathogen.

**Table 1:** The state variables of the general model of the antigen-specific T cell response in Eqs (1)–(4).

State variable	Symbol
Pathogen concentration	*B*
Number of naive T cells	*N*
Number of activated effector T cells	*A*
Number of memory T cells	*M*

**Table 2:** Parameters for the general model of the antigen-specific T cell response in Eqs (1)–(4).

Description	Symbol	Unit	Nominal Value	Likely Range
Virus replication rate	*r*	day^−1^	5	0 < *r*
Killing rate of effector T cells	*k*	day^−1^	310^−5^	0 < *k*
Production rate of naive T cells	*σ*	day^−1^	0	0 ≤ *σ*
Replication rate of naive T cells	*r*_*N*_	day^−1^	0	0 ≤ *r*_*N*_
Activation rate of naive T cells	*a*_*N*_	day^−1^	1	0 < *a*_*N*_
Death rate of naive T cells	*d*_*N*_	day^−1^	0.001	0.027 ≤ *d*_*N*_ ≤ 0.007
Proliferation rate of activated T cells	*ρ*	day^−1^	1.93	1.4 ≤≤ 3.0
Death rate of activated T cells	*d*_*A*_	day^−1^	1	0.19 ≤ *d*_*A*_ ≤ 1
Production rate of memory T cells	*m*	day^−1^	0.05	0.008 ≤ *m* ≤ 0.05
Replication rate of memory T cells	*r*_*M*_	day^−1^	0	0 ≤ *r*_*M*_
Activation rate of memory T cells	*a*_*M*_	day^−1^	1	0 < *a*_*M*_
Death rate of memory T cells	*d*_*M*_	day^−1^	0.01	0 ≤ *d*_*M*_
Immune response function	F(t))			0≤F(t))≤1
Saturation constant	*h*	PFU	10	0 < *h*

The nominal values of the parameters given in Table 2 along with the initial conditions *B*(0) = 1000; *N*(0) = 100; *A*(0) = *M*(0) = 0 are chosen to reflect the scenario of a primary specific T cell response to acute infection, see [4, 28, 29]. Similar settings have been used in other mathematical studies [3, 16].

### Variable structure control systems

**Fig 1:**
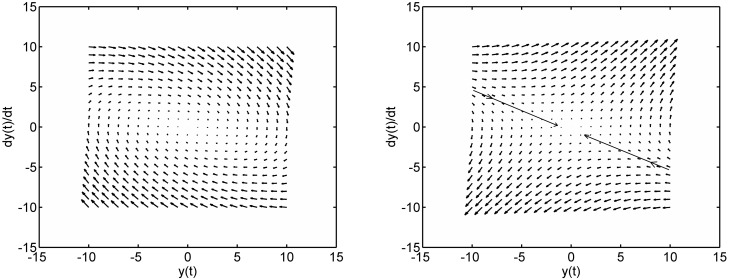
Phase portrait of two candidate fixed structure control strategies for the oscillator system with *ξ* = 0.7. Left: (a) Stable dynamic (poles at −0.35 ± 0.69*j*). Right: (b) Unstable dynamic (poles at 0.5 and −1.2). In (a), the trajectories move in a clockwise motion to reach the origin. In (b), the trajectories move away from the origin except along the asymptotes of the hyperbolic structure in the quadrants with $y\left(t\right)\frac{dy(t)}{dt}<0$.

A variable structure control system is characterised by a number of feedback control laws and a decision rule [1]. The decision rule, usually termed the switching function, has, as its input, some measure of the current system behaviour and produces as an output the particular feedback control which should be used at that instant in time [1, 30]. To present the main concepts, consider the oscillator given by

$$\begin{array}{c} \frac{d^{2}y}{dt}-\xi \frac{dy}{dt}+uy=0 \end{array}$$ (5)

where *ξ* is a constant and *u* is a gain value whose impact is to be investigated. The design objective is to force the trajectories of the second order system to reach and remain at the origin. Fig 1 shows the phase plane trajectories when *u* = 0.6 and a stable system results and when *u* = −0.6 and an unstable system results. It is seen that although the stable response is the more desirable of the two, it is slow. In comparison, the unstable response exhibits some rapid motion towards the origin in the quadrants where $y\left(t\right)\frac{dy(t)}{dt}<0$.

**Fig 2:**
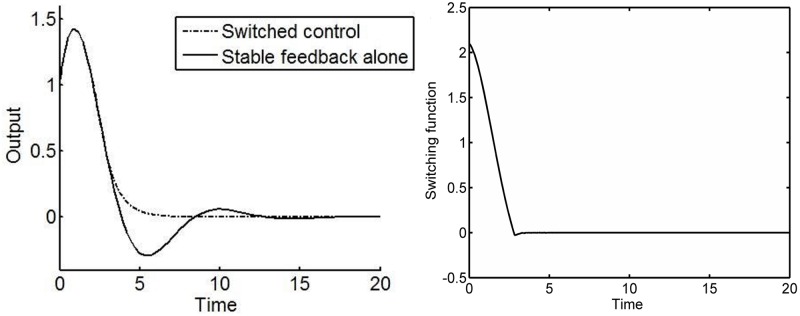
Dynamic response of the oscillator system. Left: (a) Comparison of the time evolution of the output *y*(*t*) produced by a fixed structure and a variable structure control strategy. Right: (b) Time evolution of the switching function when a variable structure control is applied to the oscillator system. In (a), the output response of the oscillator system shows that the designed switched control law yields a faster settling time than the stable feedback alone. In (b), the trajectory reaches and remains at the origin. Ideal sliding motion starts when the switching function vanishes. This indicates that the states of the system are confined to the designed sliding manifold.

The use of an unstable subsystem to impose a rapid transition of the system state is an established characteristic that is exploited in the design of VSCS [1]. To illustrate this phenomenon, define a switching function as

$$\begin{array}{c} s\left(y\left(t\right),\frac{dy(t)}{dt}\right)=cy\left(t\right)+\frac{dy(t)}{dt} \end{array}$$ (6)

with

$$\begin{array}{c} 0<c<-\frac{\xi }{2}-{\left(\frac{\xi^{2}}{4}+\alpha \right)}^{\frac{1}{2}} \end{array}$$ (7)

where *c* is a positive scalar selected to represent one of the stable poles of the system (5). A suitable VSC which combines a positive gain and a negative gain is given as:

$$\begin{array}{c} u=\left\{\begin{array}{cc} \alpha & \;\text{if}\;y\left(t\right)s(y\left(t\right),\frac{dy(t)}{dt})>0\\ -\alpha & \;\text{if}\;y\left(t\right)s(y\left(t\right),\frac{dy(t)}{dt})<0 \end{array}\right. \end{array}$$ (8)

where *α* is the chosen controller gain. The simulation results in Fig 2 use *α* = 0.6, *c* = 1.1 which corresponds to switching between the stable and unstable behaviours in Fig 1 and initial conditions $y\left(0\right)=1,\frac{dy(0)}{dt}=1$. The benefits in terms of the speed of response are immediate. It is also seen that the switching function is driven to zero. From Eq (6), it is clear that after an initial transient, the VSCS imposes a dynamic response which is entirely specified by the choice of *c*. In this particular case, the specified dynamic response corresponds to a first order dynamic system with a stable pole. Fig 2 in fact demonstrates a specific type of VSCS, so-called sliding mode control [2]. Here a variable structure control is designed to drive and then constrain the system state to lie within a neighbourhood of the switching function. Using this approach, the dynamic behaviour of the system is directly tailored by the choice of switching function with the switching function being a measure of the desired performance of the system under consideration [1, 30]. It will be seen that the ability to attain this level of performance relates directly to well-defined characteristics of the effective control effort available in the system.

**Fig 3:**
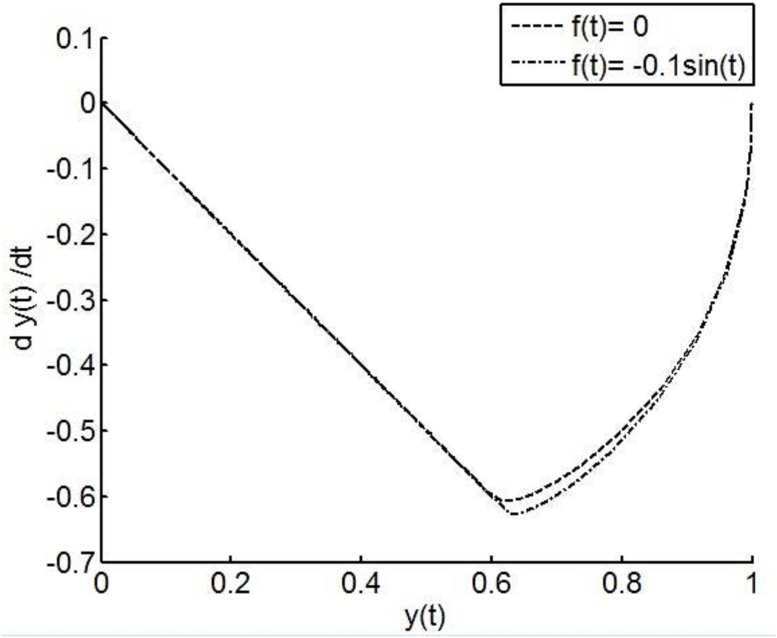
Phase plane portrait showing the response of the double integrator (*f*(*t*) = 0) and the perturbed system (*f*(*t*) = −*sin*(*y*)) with initial conditions *y*(0) = 1, $\dot{y}\left(0\right)=0$. At first, there is a transient phase, known as the reaching phase, in which the dynamics of the system move towards the selected sliding manifold. During the reaching phase, the system dynamics are affected by the perturbation signal. Following this reaching phase, a sliding mode takes place. The trajectories of the system are confined to a vicinity of the sliding manifold and move in a straight line towards the origin. It is seen that during this sliding motion, the traces of the system with and without perturbation are indistinguishable. This shows that the action of the switched control in the sliding mode cancels the effects of the perturbation signal.

The inherent robustness of VSCS with a sliding mode will now be demonstrated. Consider the following uncertain, second order system

$$\begin{array}{c} \ddot{y}=u+f\left(t\right) \end{array}$$ (9)

where *u* denotes a control action. When *f*(*t*) = 0 the dynamics Eq (9) collapse to the case of a nominal double integrator. Adopt the switching function from Eq (6). Simulations are performed for nominal conditions (*f*(*t*) = 0) and under perturbed conditions (*f*(*t*) = −0.1*sin*(*t*)) with *c* = 1 in Eq (6). In Fig 3 it is seen that in the sliding mode, when *s* = 0, the dynamics of the system are determined by the dynamics $\dot{y}=-y$, a free system where the initial condition is determined by $\left(y\left(t_{s}\right),\dot{y}\left(t_{s}\right)\right)$, where *t*_*s*_ is the time at which the sliding mode condition, *s* = 0 is reached. Identical dynamics are exhibited by both the nominal and perturbed systems and the corresponding VSCS is seen to possess very strong robustness to uncertainty and perturbations. Analysing the sliding mode dynamics is termed as solving the *existence problem* and this can be achieved using a broad range of methods such as Lyapunov techniques [1, 31]. A control to ensure the desired sliding mode dynamics are attained and maintained is sought by means of solving the *reachability problem*. A fundamental requirement to attain the desirable dynamics is that the sliding mode dynamics must be attractive to the system state and there are many *reachability conditions* defined in the literature [1, 2]. One so called reachbility condition is given by

$$\begin{array}{c} s\dot{s}<0 \end{array}$$ (10)

and it is straightforward to verify that the control

$$\begin{array}{c} u=-\dot{y}-\rho \text{sgn}\left(s\right) \end{array}$$ (11)

for *ρ* > *a*_1_ + *η* where *η* is a small positive design scalar and ‖*f*(*t*)‖ < *a*_1_ ensures the reachability condition is satisfied. Many strategies can be adopted to ensure an appropriate reachability condition is satisfied. In engineering, undesirable discontinuity of the sgn(*s*) function in Eq (11) is frequently approximated by

$$\begin{array}{c} \frac{s}{|s|+\delta } \end{array}$$ (12)

where *δ* > 0 is small.

The inherent robustness of VSCS is demonstrated by the principle of the *equivalent injection*. Assume the sliding mode condition holds and differentiate Eq (6) with respect to time. Substituting from the system dynamics Eq (9) yields

$$\begin{array}{ccc} \dot{s}\left(t\right) & = & c\dot{y}+\ddot{y}\\ & = & c\dot{y}+u_{eq}+f\left(t\right)\\ & = & 0 \end{array}$$ (13)

**Fig 4:**
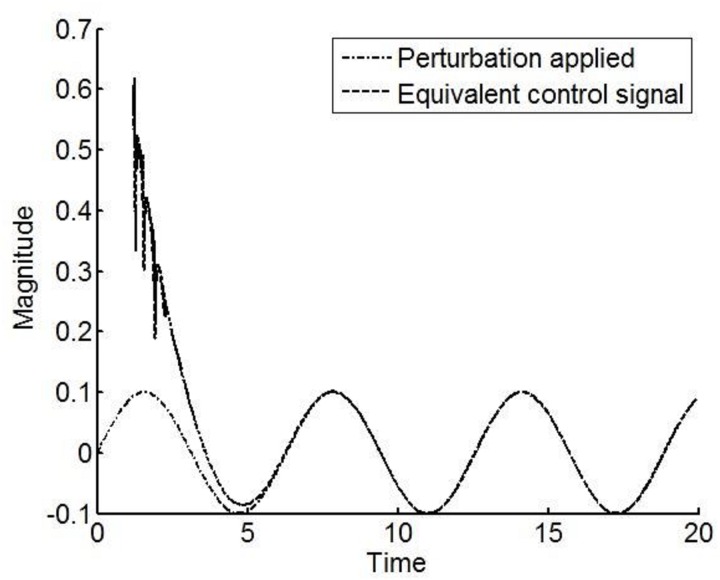
Time evolution of the equivalent control signal and the perturbation signal. This shows the relationship between the applied control signal and the external perturbation signal once the sliding mode is reached. The response of the equivalent control signal at earlier time points is not provided because by definition, the equivalent control occurs only after the sliding mode takes place. Importantly, this graph shows that when a sliding mode is enforced, the equivalent control can be used to reconstruct the dynamics of the perturbation signals implicit in the channel where the control signal is applied, the so-called matched uncertainty.

The effective control action has been referenced as *u*_*eq*_ in Eq (13) because it is not the applied control but represents the effective action of the applied discontinuous control once the sliding condition *s* = 0. The robustness of the VSCS is effectively demonstrated from Eq (13) where it is seen that the effect of the uncertain terms on the dynamics are effectively cancelled out, provided the reachability condition Eq (10) holds and the sliding mode is reached. For the simulation study in Fig 3, the applied control signal is seen to effectively reconstruct the unknown external perturbation in Fig 4.

Matlab scripts to reproduce the simulations and results can be found in S1 File.

## Results

### The dynamics of the specific T cell response as a VSCS

**Fig 5:**
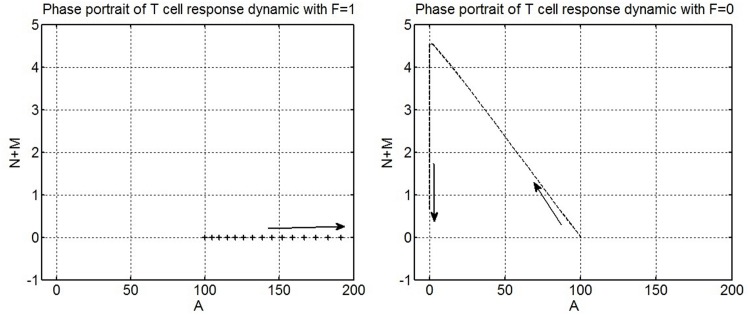
Phase portrait analysis of the T cell population dynamics produced by an immune response function with a fixed control structure. Left: (a) Phase portrait of T cell population dynamics with F(t))=1. Right: (b) Phase portrait of T cell population dynamics with F(t))=0. In (a), the trajectory of the T cell response generated by F(t))=1 is shown. This induces unstable dynamics and a motion away from the origin. The trajectory depicts an exponential and unbounded expansion of activated T cells. In (b) the trajectory of the T cell response generated by F(t))=0 is seen. The trajectory exhibits a stable motion towards the origin. This motion depicts the contraction of the population of activated T cells and the formation of memory T cells.

The analysis in this section is conducted to test the hypothesis that a variable structure control paradigm is appropriate to model the immune response. Simulation of the system Eqs (1)–(4) reveals that the magnitude of the steady-state population of the pathogen and antigen-specific T cells is influenced by the function F(t). The phase portrait of the population dynamic of T cells with an activation function having a single structure with either F(t)=1 or F(t)=0 is shown in Fig 5. Since the system depicts the dynamics of biological populations, the motion of the trajectories is confined to the positive quadrant in the results presented.

Fig 5 demonstrates that the system exhibits stable behaviour, which shapes the contraction phase and the differentiation of activated T cells into memory T cells, when F(t)=0 and unstable behaviour, corresponding to exponential growth of activated T cells, when F(t)=1

**Fig 6:**
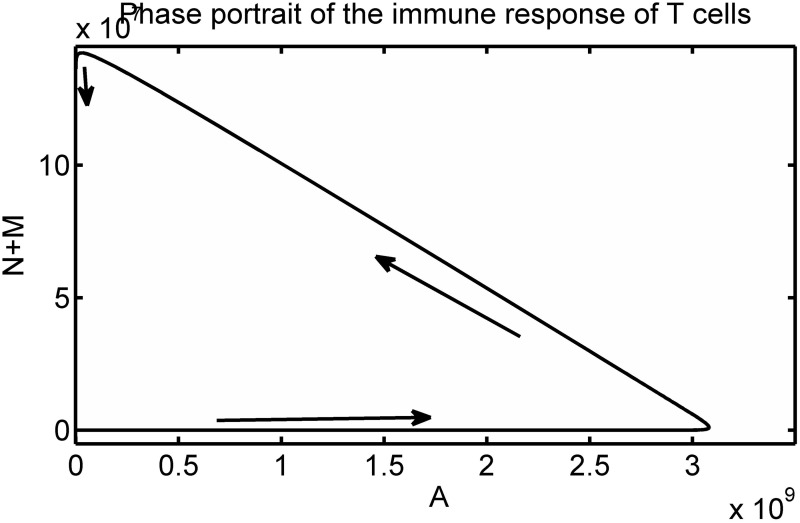
Phase portrait of the trajectories of the immune system Eqs (1)–(4) with the antigen-dependent activation function Eq (14).

It has been seen that the immune system dynamics Eqs (1)–(4) exhibit stable and unstable sub-systems and simulation of an appropriate switching strategy will now be performed. Noting the similarity with the control strategy from Eq (12), the saturation function studied in [3] is adopted as the candidate activation function:

$$\begin{array}{ccc} F_{1}\left(t\right)= & F(B)= & \frac{B}{h+B} \end{array}$$ (14)

where *h* > 0 is a saturation constant. The phase portrait of the corresponding immune response in Fig 6 demonstrates that the chosen antigen-dependent activation function from Eq (14) provides a smooth switch between the different immunological dynamics of the immune response.

**Fig 7:**
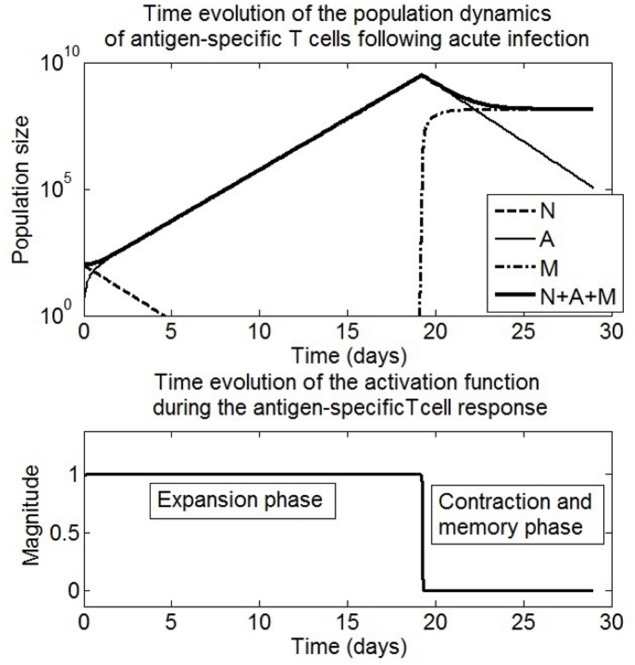
Simulation of the scenario of T cell population dynamics following acute infection. Left: Time evolution of the population dynamics of the antigen-specific T cell response. Right: the time evolution of the antigen-dependent activation function Eq (14). Following infection, the magnitude of the activation function is at a maximum. This induces the reduction of the state *N* and an increase of the state *A*. The state variable *N* decreases because naive T cells become activated T cells. The state variable *A* increases due to the expansion of the population of activated T cells. When the magnitude of the immune response function falls to zero, the expansion phase is interrupted. There is production of memory T cells, the state *M* increases whilst the activated T cells undergo contraction i.e the state *A* decreases. Consequently, the activation function prescribes the variation over time of the total population of the antigen -specific T cells (*N* + *A* + *M*) following infection.

Fig 7 displays the variation over time of the population size of naive, activated and memory T cells generated by the immune response function Eq (14). The time evolution of the total number of antigen-specific T cells following infection is shown because this is a typical output measurement presented by kinetic studies of the specific T cell response in mice models, see [4, 7, 29]. The population dynamics of the specific T cell response shown in Fig 7 are similar to the ones observed in experimental studies. A rapid increase in the total antigen-specific T cell population from an initial small number is observed, followed by a sudden decay of this population. This dynamical behaviour represents the expansion, contraction and memory phase of the specific T cell response observed in experimental studies [4, 10, 29].

### A sliding mode control perspective

Consider the following switching function

$$\begin{array}{ccc} s_{1} & = & A \end{array}$$ (15)

which has been selected because the manifold *s*_1_ = *A* = 0 is associated with the absence of an ongoing immune response and a healthy state. Application of the reachability condition (10) shows that the system reaches and remains at the healthy state if:

$$\begin{array}{cc} s_{1}A\left(F\left(t\right)\left(a_{N}N+a_{M}M+\rho A\right)-\left(1-F\left(t\right)\right)\left(mA\right)-d_{A}A\right) & <0 \end{array}$$ (16)

**Fig 8:**
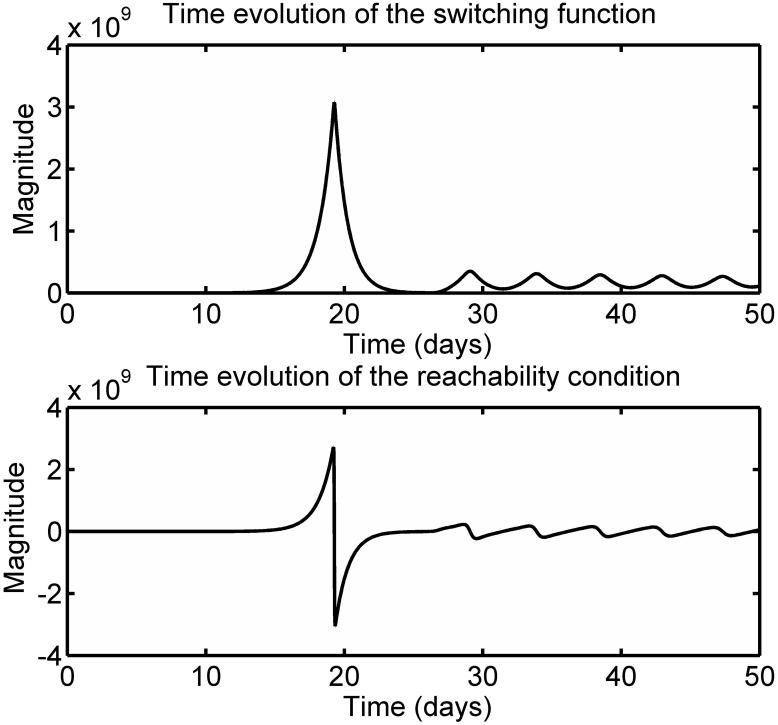
Time evolution of the sliding surface and reachability condition for the immune system Eqs (1)–(4) with the antigen-dependent activation function (14).

This inequality reinforces that the candidate immune response function F(t) plays a major role in governing the dynamical changes occurring in the population of activated T cells. Eq (16) illustrates analytically the switching conditions governing the activation and contraction of the antigen-specific T cell response. Fig 8 displays the time evolution of the sliding surface and reachability condition for the immune system Eqs (1)–(4) with the antigen-dependent activation function Eq (14). Considering both Figs 7 and 8, it can be deduced that the magnitude of Eq (14) during the expansion phase leads to the reachability condition being positive i.e $s_{1}\frac{ds_{1}}{dt}>0$. Although the trajectory of Eq (15) moves away from the manifold *s*_1_ = *A* = 0, this transient motion is desirable in order to increase the number of activated T cells to a sufficient level to contain the infection. After a finite time period, the manifold *s*_1_ = *A* = 0 is rendered attractive and the trajectory of Eq (15) moves towards this manifold because the magnitude of the candidate immune response function Eq (14) satisfies the reachability condition Eq (16). The sliding mode reachability condition thus offers an analytical mechanism to monitor and evaluate the immune response.

Note that in Fig 8, the state of the system does not remain on the desired sliding surface *s*_1_ = *A* = 0. Therefore, the system Eqs (1)–(4) does not reach the healthy state. Infact the model Eqs (1)–(4) provides a good description of the acute infection dynamics up to day 28 [3]. Following day 28, the candidate immune response function performs a sequence of activation and inhibition of the immune response which causes the oscillations observed in Fig 8. This is not as expected from biology. In essence, F(B) does not vanish due to the fact that the state variable *B* does not decay to zero following the primary immune response. As a result, F(B) does not allow the reachability condition (16) to hold. In this case, the reachability analysis explains why the model is not valid in the steady state. The way in which the pathogen dynamic is modeled is not appropriate to model acute infection due to the fact that it does not allow the pathogen *B* to vanish following the immune response [4]. Consequently, subsequent results focus on the first 28 days post-infection in the rest of the paper.

### Robustness of the T cell response

In immunology, it has been observed that some pathogen induce undesirable proliferation or death of activated T cells after infection to perturb the immune system [3–5, 32]. To simulate this phenomenon, the expression for the population dynamic of activated T cells Eq (3) is revised:

$$\begin{array}{ccc} \frac{dA}{dt} & = & F\left(t\right)\left(a_{N}N+a_{M}M+\rho A+mA\right)-\left(m+d_{A}\right)A+d_{u} \end{array}$$ (17)

where the parameter *d*_*u*_ encompasses biological disturbances affecting the variation over time of the population of activated T cells. The impact of these perturbations on the T cell response is investigated using a reachability analysis. The reachability condition becomes

$$\begin{array}{cc} A\left(F\left(t\right)\left(a_{N}N+a_{M}M+\rho A+mA\right)-\left(m+d_{A}\right)A+d_{u}\right) & <0 \end{array}$$ (18)

This inequality shows that perturbations affecting the population of activated T cells act directly on the channel in which the immunological feedback F(t) governing the T cell response occurs. Therefore, biological perturbations such as down-regulation signals from pathogen which affect the dynamics of activated T cells can be classified as matched uncertainty. From a sliding mode control perspective, this implies that when the magnitude of *d*_*u*_ is sufficiently small so that the sign of $\frac{ds_{1}}{dt}$ is preserved, the dynamics of the T cell response is preserved. The sign of Eq (18) is insensitive to changes in the biological rates *d*_*A*_ and *ρ* in Table 1. Therefore, the T cell response is robust to variation in these biological rates.

The inherent robustness to perturbations can be analysed using the principle of the equivalent injection. From Eq (18), it follows that in the sliding mode

$$F{\left(t\right)}_{eq}=-\frac{d_{u}}{a_{N}N+a_{M}M}$$ (19)

and it is seen by applying the equivalent injection signal from Eq (19) in Eq (17) that the action of the immune response cancels the effect of the disturbance signal in the sliding mode.

**Fig 9:**
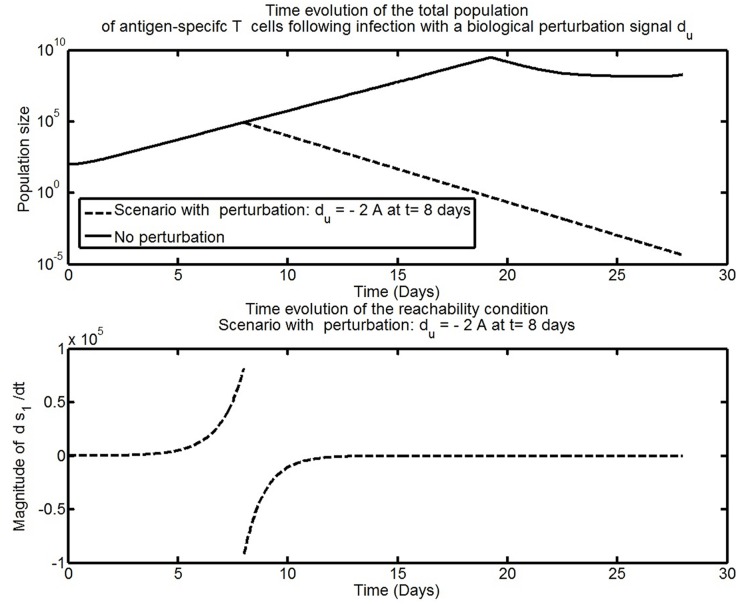
Time evolution of the reachability condition in the presence of the perturbation *d*_*u*_ = −2*A* for *t* > 8 and *d*_*u*_ = 0 otherwise.

The simulation results shown in Fig 9 mimic the case in which the population dynamic of activated T cells is subject to virus-induced perturbations during the immune response. The initiation of a perturbation defined as *d*_*u*_ = −2*A* interrupts the expansion phase and reduces dramatically the population of activated T cells. This phenomenon is often observed during chronic infection [4, 27]. The time evolution of the reachability condition shows that the immunological feedback sustaining the expansion of activated T cells cannot maintain $s_{1}\frac{ds_{1}}{dt}>0$ when the perturbation *d*_*u*_ = −2*A* occurs. Considering the expression of the reachability condition in Eq (18), a candidate solution to achieve robust expansion dynamics is to increase the proliferation rate of activated T cells, *ρ*, to a sufficient level to keep $s_{1}\frac{ds_{1}}{dt}>0$. For completeness, the reduced order dynamics in the sliding mode are analysed. Assume a sliding mode has been reached so that *A* = 0. Consider the candidate Lyapanov function *V*(*N*, *M*) for the remaining dynamics given by

$$\begin{array}{ccc} V(N,M) & = & N+M \end{array}$$ (20)

Since *N* ≥ 0 and *M* ≥ 0, the Lyapanov function *V*(*N*, *M*) is positive definite as required [31]. The temporal derivative of Eq (20) is

$$\begin{array}{ccc} \frac{dV(N,M)}{dt} & = & -\left(a_{N}N+a_{M}M\right)F{\left(t\right)}_{eq}-d_{N}N-d_{M}M \end{array}$$ (21)

**Fig 10:**
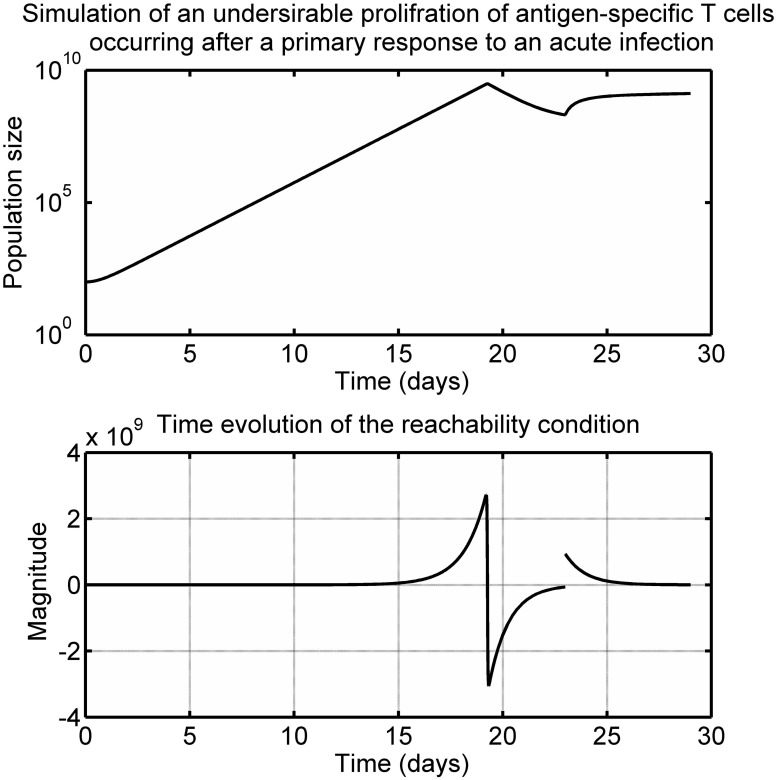
Time evolution of the total population of antigen-specific T cells following infection with an undesirable proliferation signal *d*_*u*_ at day 23.

From Eq (19), the dynamics in the sliding mode are a function of the disturbance. For the case of a vanishing perturbation signal such as *d*_*u*_ = −2*A*, it is clear that the corresponding equivalent injection becomes zero and from Eq (21) the dynamics are stable. It is also clear from Eq (21) that non-vanishing perturbation signals can affect the stability of the immune system in the steady state. Consider the scenario of an undesirable expansion of activated T cells caused by some unwanted triggers such as a super antigen [5, 33], see Fig 10. When the unwanted proliferation signal *d*_*u*_ occurs, the reachability condition for the decay of activated T cells fails and the sliding motion towards *s*_1_ = *A* = 0 is interrupted.

### Dynamical requirements for effective CD4+ T cell help: a variable structure control approach

Consider the following closed-loop dynamics to represent the memory CD8+ T cell response following chronic LCMV infection in mice:

$$\begin{array}{ccc} \frac{dA}{dt} & = & F\left(t\right)\left(a_{M}M+\rho A\right)-\left(1-F\left(t\right)\right)\left(m+d_{A}\right)A-d_{u}+u_{h}\left(t\right) \end{array}$$ (22)

$$\begin{array}{ccc} \frac{dM}{dt} & = & \left(1-F\left(t\right)\right)mA-a_{M}F\left(t\right)M-d_{M}M \end{array}$$ (23)

This representation is chosen to make use of existing parameter values estimated from kinetic data on LCMV-specific memory CD8+ T cells following chronic LCMV infection [4, 27]. The term *d*_*u*_ represents virus-induced perturbations on the cellular dynamics of the immune response of memory CD8+ T cells during chronic infection. The findings in [4] show that large virus titers and antigen persistence observed during chronic LCMV infection lead to a reduction in the proliferation rate and population size of secondary effector LCMV-specific CD8+ T cells as well as early contraction of the response. Let *u*_*h*_(*t*) be the mechanism by which the LCMV-specific CD4+ T cell response helps the expansion of LCMV-specific memory CD8+ T cells activated following chronic infection. Here, the mechanism of CD4+ T cell help i.e *u*_*h*_(*t*) is analyzed as a control input acting on the dynamics of activated CD8+ T cells. This formulation is motivated by the fact that results in [4] have demonstrated that CD4+ T cell help is a suitable means to improve the response of memory CD8+ T cells.

Using the VSC approach, it follows that to sustain the expansion of secondary effector CD8+ T cells, the mechanism of CD4+ T cell help must satisfy the following dynamical condition:

$$\begin{array}{c} \frac{dA}{dt}=F\left(t\right)\left(a_{M}M+\rho A\right)-\left(1-F\left(t\right)\right)\left(m+d_{A}\right)A-d_{u}+u_{h}\left(t\right)>0 \end{array}$$ (24)

This demonstrates that the mechanism of CD4+ T cell help is a suitable treatment strategy to yield a robust expansion of secondary effector CD8+ T cells following chronic infection. The inequality Eq (24) indicates that the magnitude of CD4+ T cell help must be sufficiently large despite the effects of *d*_*u*_, which represents down-regulation signals imposed by large virus titers and antigen persistence. This dynamical condition must hold independent of any explicit mathematical expression chosen to model the immune response function F(t), the virus-induced parameter perturbations and the CD4+ T cell help mechanism *u*_*h*_(*t*). Eq (24) provides a time varying threshold for the CD4+ T cell help mechanism to be effective in sustaining the expansion of secondary effector CD8+ T cells during chronic infection. For simulation, the following time-based activation function is considered

$$\begin{array}{ccc} F(t)=1 & \text{if} & t_{on}\leq t\leq t_{off}\\ F(t)=0 & & \text{otherwise} \end{array}$$ (25)

where experiment suggests

- *t*_*on*_: the time (in days) at which the expansion of LCMV-specific CD8+ T cells starts
- *t*_*off*_: the time (in days) at which the expansion stops and the contraction and memory phase of the response starts

**Fig 11:**
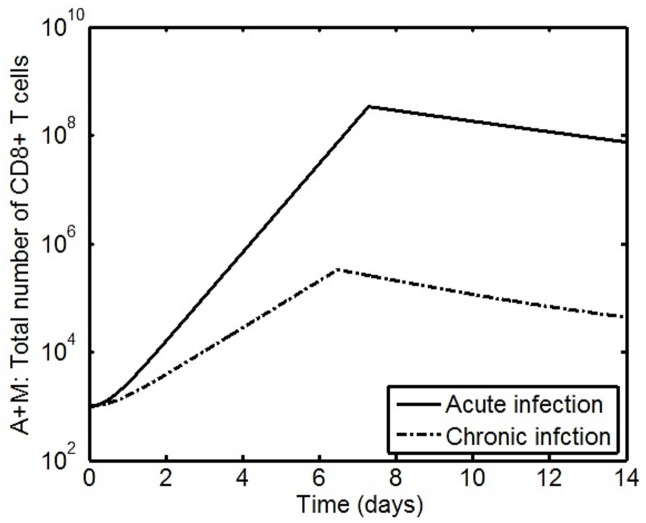
Time evolution of the population dynamics of LCMV specific memory CD8+ T cells following acute and chronic infection and no CD4+ T cell help. Following infection, memory CD8+ T cells expand in large numbers and undergo a contraction phase after a few days. The duration of the expansion phase during chronic infection is shorter than during acute infection. The number of CD8+ T cells produced during the expansion phase is smaller in chronic infection when compared to acute infection. The decay of the number of CD8+ T cells is faster in the case of chronic infection when compared to acute infection.

**Fig 12:**
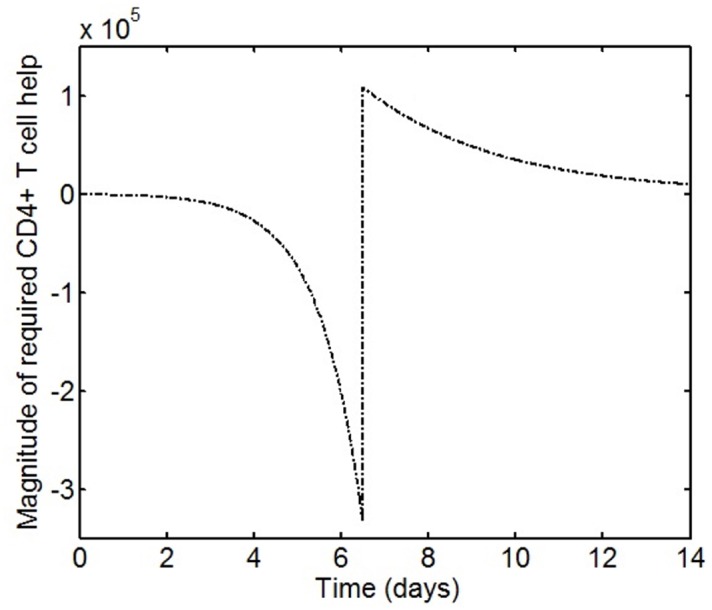
Time evolution of the level of CD4+ T cell help reqired to support an effective secondary effector CD8+ T cell response following chronic infection. Eq (24) is used to extract the variation over time of the level of CD4+ T cell help from the memory CD8+ T cell response dynamics. Note that the trace defines the lower bound of the required help. The negative phase corresponds to the expansion phase and the positive phase corresponds to the contraction phase. The magnitude of the help required to sustain the memory CD8+ T cell response following chronic infection is small during the expansion phase and increases due to the early contraction.

The settings in Table 3 are chosen to simulate the cellular dynamics of the response of memory CD8+ T cells specific to the LCMV GP33 epitope following infection where no CD4+ T cell help is present. The parameter estimation work in [27] has shown that the effects of chronic infection are reflected in the variations in the values of the biological rates *m*, *d*_*A*_, *t*_*off*_. In [27], it was assumed that the net expansion rate *ρ* of memory CD8+ T cells after acute and chronic infection is the same. Nonetheless, from the experimental results in [4], it is hypothesized that the value of the expansion rate *ρ* will also be reduced. Fig 11 shows that the dynamics produced by the model Eqs (22)–(23) using the settings in Table 3 are similar to the memory T cell response following LCMV infection observed in experiments [4]. Of interest, the secondary effector CD8+ T cell response in the simulation has a lower number of cells at day 6.5 This validates the hypothesis that the net expansion rate of memory CD8+ T cells is reduced after chronic infection. This reduced expansion along with the early contraction show that the chronic infection without CD4+ T cell help is impaired. Fig 12 shows the level above which the magnitude of CD4+ T cell help must be to sustain the expansion phase of the immune response which is generated from Eq (24). The threshold is initially smaller because, despite the virus-induced perturbation due to chronic infection, memory CD8+ T cells mount a short expansion phase. Further, the threshold increases when the presence of a high virus load and antigen persistence induces early contraction of the immune response. Hence, the VSC approach provides a mechanism to monitor the level of CD4+ T cell help required. From the perspective of control engineering, analysis suggests that reducing the value of *ρ* during chronic infection leads to a reduction in the magnitude of this unstable pole during the expansion phase. As a result, the speed of response (proliferation) is decreased. Further, early contraction produces stable poles (−(*m* + *d*_*A*_) which interrupts the expansion. Consequently, it is possible to achieve a better outcome in terms of T cell help by increasing the proliferation rate of secondary effector CD8+ T cells and/or the duration of the expansion.

**Table 3:** Simulation setting for modelling the LCMV-specific memory CD8+ T cell response without CD4+ T cell help.

Symbol	Acute infection	Chronic infection
*A*(0)	0	0
*M*(0)	1000	1000
*ρ*	1.89	1
*d*_*A*_	0.23	0.31
*m*	0.004	0.017
*a*_*M*_	1	1
*d*_*M*_	0	0
*t*_*on*_	0	0
*t*_*off*_	7.3	6.5

**Fig 13:**
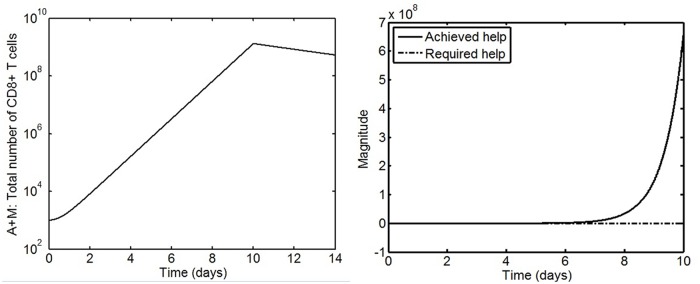
Simulation of the time evolution of secondary CD8+ T cells with CD4+ T cell help *u*_*h*_(*t*) = 0.5 and *t*_*off*_ = 10 during chronic infection. Top: (a) Time evolution of the population dynamics of LCMV specific memory CD8+ T cells following chronic infection and CD4+ T cell help. Bottom: (b) Time evolution of the level of CD4+ T cell help. The action of CD4+ T cell help increases the number of CD8+ T cells as well as the duration of the expansion. Further, the magnitude of CD4+ T cell help is proportional to the number of secondary effector CD8+ T cells. The achieved level of CD4+ T cell help is higher than that required throughout the simulation.

Consider the following candidate control function for CD4+ T cell help *u*_*h*_(*t*) = *ρ*_*h*_*A*. Using Eq (24), effective CD4+ T cell help is designed as follows:

$$\begin{array}{cc} \left(1-F\left(t\right)\right)\left(m+d_{A}\right)A+d_{u}-F\left(t\right)\rho A & <\rho_{h}A \end{array}$$ (26)

since *M* → 0 during the expansion. Consequently, when Eq (26) is satisfied, CD4+ T cell help ensures robust expansion of secondary effector CD8+ T cells. Simulation results in Fig 13 illustrate the case of CD4+ T cell help during chronic infection. The addition of CD4+ T cell help improves the kinetics of the immune response. There is an increase in the speed of the proliferation as well as the number of CD8+ T cells. The designed CD4+ T cell help is seen to sustain the expansion of secondary effector CD8+ T cells despite the effects of chronic infection.

The experimental results of [4] show that with the same number of CD8+ T cells at day 0, the secondary effector CD8+ T cell response with the action of CD4+ help leads to a higher number of CD8+ T cells at day 6.5, see figure 5 in [4]. This implies that the action of CD4+ help influence the speed of response and increases the net expansion rate of CD8+ T cells. Further, it is assumed that CD4+ T cell help will also increase the duration of the expansion because the number of CD8+ T cells at day 6.5 is not enough to clear the infection due to the observed large virus load. Hence, CD4+ T cell help operates as a control action which influences both the poles and the timings of the immune response dynamics of memory CD8+ T cells following chronic infection.

## Discussion

The variable structure control paradigm is seen to provide an analytical framework with which to articulate the characteristics of the immune response function for the model of the antigen-specific T cell response Eqs (1)–(4). It has been demonstrated that the dynamical behaviour of the antigen-specific T cell response naturally incorporates stable and unstable sub-systems. The unstable subsystem has a single steady-state T cell population located at the trivial equilibrium and stability analysis reveals that this equilibrium is unstable. As a result, the expansion phase is underpinned by an unstable positive feedback loop which enables the trajectories of the system to rapidly move away from a low steady-state population [7, 14, 23] and contributes to an increase in the population size of activated T cells as required to suppress the pathogen. This unstable dynamic is desirable from the view-point of immunology for a short time period but prolonged expansion of activated T cells may lead to disease or the damage of healthy cells [14, 33]. The second sub-system is stable and the T cell population dynamic exhibits a stable motion towards the trivial equilibrium. This shapes the contraction phase and the differentiation of activated T cells into memory T cells.

Different candidate immune response activation functions have been proposed in the literature [3, 9] with the aim of providing an appropriate switching mechanism which engenders a realistic immune response population dynamic and a realistic transient between the different phases of the immune response [9, 16]. From the perspective of variable structure control, it is well established that there are a number of candidate switched control strategies which will yield a given performance.

Here Eq (14), adopted from the domain of immunology [3], delivers a smooth transition between the extremes of F(t)=1 and F(t)=0. In fact, the switching function Eq (14) correlates with the smoothed unit-vector nonlinearity which is frequently used to implement sliding mode control laws in the domain of engineering [2, 30] in order to overcome the undesirable impact of prolonged switching and ensure a smooth control signal is applied to the plant.

The analysis of the simulation of the model Eqs (1)–(4) with Eq (14) reveals that the model appropriately captures the dynamics of the T cell response to acute infection for a given period of time. This limitation is due to the mathematical relationship between the pathogen dynamic Eq (1) and the candidate antigen-dependent immune response function Eq (1). On the one hand, the mathematical expression of Eq (1) has an unstable pole i.e *r* > 0 at the healthy state where *B* = 0. This feature allows the model to reproduce the rapid growth of the pathogen in the early days post infection but also prevents the model exhibiting realistic kinetics of pathogen decay and clearance observed after acute infection [4]. On the other hand, the candidate antigen-dependent immune response function Eq (1) solely relies on the pathogen dynamics to provide the required switch in the feedback regime governing the population dynamics of T cells. Although this simple expression delivers reasonable results, it is clear that the connection between the antigen stimulation and T cell response dynamics is more sophisticated. Consequently, in other studies it is important to determine the boundaries within the model match experimental observation and to investigate a sensible relationship between the kinetics of the pathogen concentrations and the kinetics of T cell populations.

A manifold which, if attained, is consistent with achieving a healthy steady-state has been defined. It has been shown that conditions which ensure the manifold is reached can be used to highlight the switching conditions that are required to ensure an appropriate expansion and contraction phase of the population of activated T cells. The candidate immune response function needs to enforce switching in order to allow the production of activated T cells to both respond to infection and to induce the decay of activated T cells following the clearance of that infection. The “programmed” response of CD8+ T cells investigated in [3, 9, 21, 34] has been interpreted as a robust variable structure control law defining the population dynamic of the specific CD8+ T cell response. Thus, the specific T cell response to infection can be studied as a system in which the structure and the dynamic of the inherent immunological control feedback changes purposefully to achieve the robust immunological performance observed in experiments. Hence, the analysis of the specific immune response of T cells using control theory opens a new framework to evaluate immunological dynamics and might contribute to strengthen current findings in immunology.

It is shown using a reachability analysis and the principle of the equivalent injection that as long as the immunological feedback sustaining the clonal expansion or the contraction has sufficient magnitude, the effect of pathogen-induced perturbations are overcome. This supports the results of experimental work in which the qualitative behaviour of the dynamic response of naive CD8+ T cell response is preserved despite the effects of high virus load, changes in virus strains and initial dose of infection [4, 10, 16]. The reachability analysis shows that the antigen-specific response of T cells exhibits some intrinsic robustness to pathogen-induced perturbations such as down-regulation signals due to high viral load. It should be noted that this reachability condition provides a dynamical condition which is dependent on the states of the system to assess the immune response. This is in contrast to some steady-state analysis that is currently performed to analyse the dynamics of the specific T cell response following infection such as that based on the reproductive ratio [35, 36].

The variable structure control paradigm supports results on the design of successful vaccines for chronic infections. The VSC approach confirms that the CD4+ T cell help mechanism is a suitable means to render the expansion dynamics of secondary effector CD8+ T cells robust to the down-regulation signals from high viral load and antigen persistence. Analysis of the switched control is used to formulate a dynamical condition to maintain the expansion of activated LCMV-specific memory CD8+ T cells following chronic infection. The time evolution of this dynamical condition during chronic infection provides a mechanism to quantify the level of CD4+ T cell help required to sustain the proliferation dynamic of memory CD8+ T cells. The findings indicate that the required magnitude of CD4+ T cell help must change over time.

Results on the dynamics of the CD4+ T cell help mechanism motivated a literature survey to explore other cases in which the expansion dynamics of T cell populations are mutually supportive. It has been found in [37] that recent experimental studies on the primary T cell response to acute infection suggest that a portion of adaptive regulatory T cells support the expansion dynamics of CD4+ T cells when there is a lack of cytokine IL-2. Interestingly, this mechanism is modelled by a saturation function which yields a step-like change in the magnitude of this support mechanism in the days following infection. Hence, the predicted dynamics of the CD4+ T cell help mechanism to sustain CD8+ T cell expansion during chronic infection seems realistic. Further experimental studies will be required to validate this insight and simulation tools such as suggested in this paper may provide a useful contribution to experimental design.

Collectively, the work in this paper demonstrates that variable structure control theory can be a useful modelling and simulation tool in biology.

## Supporting Information

S1 File Matlab codes. Matlab scripts to reproduce the analysis and to generate the figures can be found in this file.
(ZIP)
